# Diversity of polypores in the Dominican Republic: *Pseudowrightoporia dominicana* sp. nov. (Hericiaceae, Russulales)

**DOI:** 10.3897/mycokeys.34.25371

**Published:** 2018-05-16

**Authors:** Alfredo Vizzini, Claudio Angelini, Cristiano Losi, Enrico Ercole

**Affiliations:** 1 Department of Life Sciences and Systems Biology, University of Torino, Viale P.A. Mattioli 25, I-10125, Torino, Italy; 2 Herbario Jardín Botánico Nacional Dr. Rafael Ma. Moscoso, Apartado 21-9, Santo Domingo, Dominican Republic; 3 Via Cappuccini 78, I-33170 Pordenone (PN), Italy; 4 Canaregio, 3608 – I-30121 Venezia, Italy

**Keywords:** Basidiomycota, Agaricomycetes, Caribbean Islands, Polypores, Phylogeny, Taxonomy

## Abstract

The new species *Pseudowrightoporia dominicana* is described from the Dominican Republic based on morphological and molecular data (nrITS and nrLSU sequence analyses). It is mainly characterised by pileate basidiomata with a bright pinkish context and a di-trimitic hyphal system. Phylogenetically, it is sister to the African species *P. gillesii* and to the Asiatic *P. japonica*.

## Introduction

The genus *Wrightoporia* Pouzar, typified with *W. lenta* (Overh. & J. Lowe) Pouzar (Pouzar 1966), is traditionally characterised by resupinate to pileate basidiomata, annual to perennial habit, small to medium pores and cottony to hard texture. Hyphal system monomitic to di-trimitic, generative hyphae clamped or rarely with simple septa, skeletal hyphae dextrinoid, partially dextrinoid (only in the tubes) or not dextrinoid. Basidiospores small, cylindrical to globose, smooth to finely asperulate, amyloid (Ryvarden 1982, 2016; David and Raichenberg 1987, Stalpers 1996, Núñez and Ryvarden 2001, Hattori 2008). To date, there are 52 species transferred to or described in the genus (Index Fungorum 2018). This genus belongs to the Hericiaceae, in the Russulales (Larsson and Larsson 2003, Chen et al. 2016).

Chen et al. (2016), on the basis of combined nrITS/nrLSU phylogenetic analyses and morphological data, indicated that the genus *Wrightoporia*, as currently circumscribed, is strongly polyphyletic and recognised six clades in *Wrightoporia* s.l. Consequently, species previously treated in *Wrightoporia* were transferred to *Amylonotus* Ryvarden, *Amylosporus* Ryvarden and to the three new genera *Larssoniporia* Y.C. Dai, Jia J. Chen & B.K. Cui, *Pseudowrightoporia* Y.C. Dai, Jia J. Chen & B.K. Cui and *Wrightoporiopsis* Y.C. Dai, Jia J. Chen & B.K. Cui. In particular, the genus *Pseudowrightoporia* was established by Chen et al. (2016) to accommodate *Wrightoporia cylindrospora* Ryvarden (the generic type), *W. japonica* Núñez & Ryvarden, *Pseudowrightopora crassihypha* Y.C. Dai, Jia J. Chen & B.K. Cui, *P. hamata* Y.C. Dai, Jia J. Chen & B.K. Cui and *P. oblongispora* Y.C. Dai, Jia J. Chen & B.K. Cui, species causing white rot and mostly characterised by soft corky to corky basidiomes, shining pores, dimitic hyphal structure with clamped generative hyphae and skeletal hyphae, ellipsoid, finely asperulate and amyloid basidiospores and a subtropical to tropical distribution. Based only on these morphological characteristics, the following species were transferred to *Pseudowrightoporia*: *Wrightoporia africana* Johans. & Ryvarden, *W. aurantipora* T. Hatt., *W. gillesii* A. David & Rajchenb., *W. solomonensis* (Corner) T. Hatt. and *W. straminea* T. Hatt.

During the species diversity study of wood-inhabiting macromycetes in the Dominican Republic, a pileate *Pseudowrightoporia* was discovered. The aim of this investigation was to identify and to analyse the *Pseudowrightoporia* specimens using both morphological and molecular techniques.

## Materials and methods

### Morphology

Photographs of fresh basidiomata were taken *in situ* by a Nikon Coolpix 8400 digital camera and then dried, while the photos of the microscopical structures were obtained through a Olympus BH-2 light microscope and a Nikon D7100 digital camera. For microscopical analysis, tiny fragments from dried material were mounted in Melzer’s anionic reagent for testing amyloid and dextrinoid reactions of spores and other microscopical elements. All microscopic measurements were carried out with a ×1000 oil immersion objective. Basidiospores were measured from hymenophores of mature basidiomes, dimensions are given as: (minimum–) average minus standard deviation – *average* – average plus standard deviation (–maximum) of length × (minimum–) average minus standard deviation – *average* – average plus standard deviation (–maximum) of width; Q = (minimum–) average minus standard deviation – *average* – average plus standard deviation (–maximum) of the length/width ratio. Spore statistics were produced using R version 3.4.4 (R Core Team 2018). Herbarium acronyms follow Thiers [2018, continuously updated] with the exception of ANGE that refers to the personal herbarium of C. Angelini.

### DNA extraction, PCR amplification and DNA sequencing

**Figure 1.**
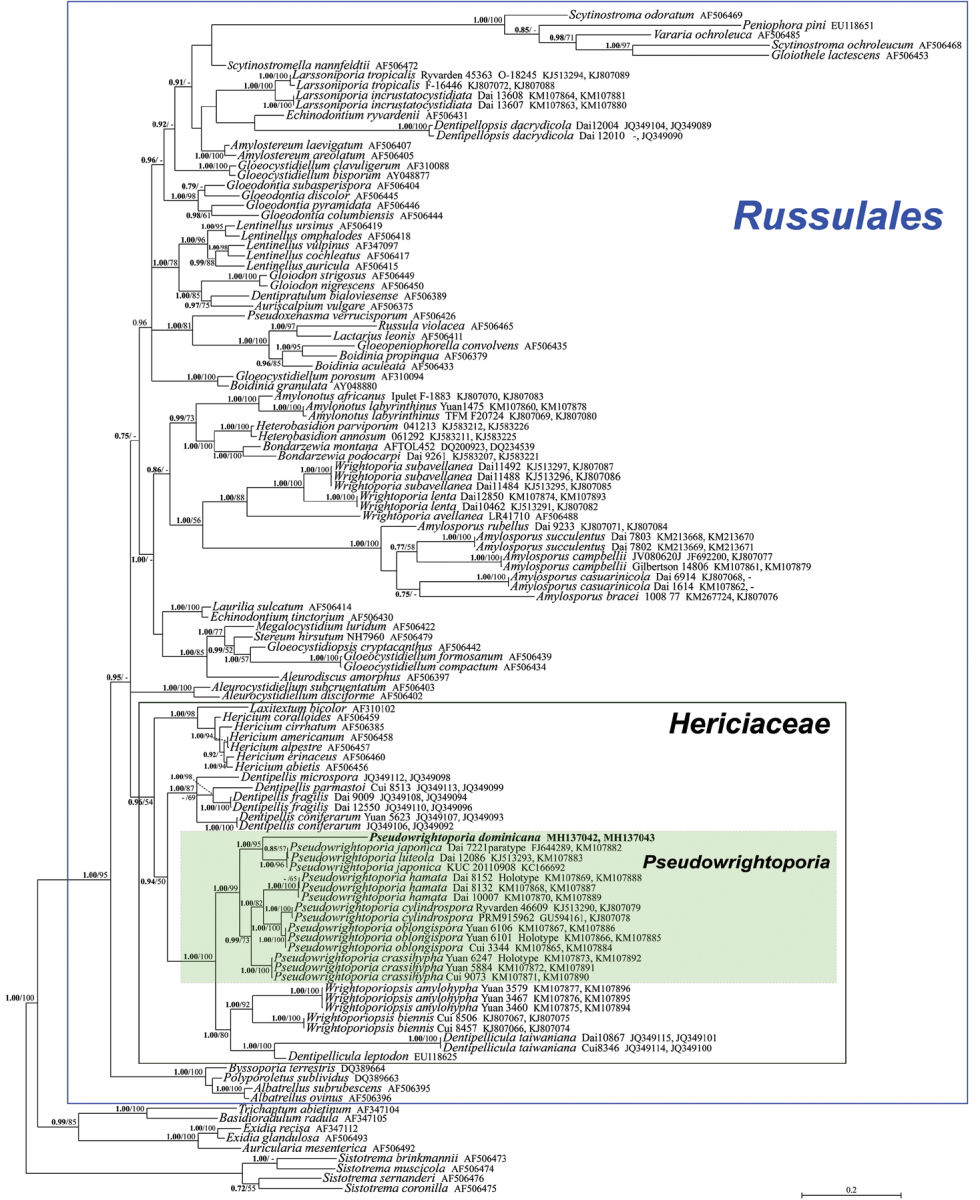
Bayesian phylogram obtained from the combined nrITS-nrLSU sequence alignment of *Russulales* taxa selected according to Chen et al. (2016). *Sistotrema brinkmannii*, *S. coronilla*, *S. muscicola* and *S. sernanderi* were used as outgroup taxa. Values for clades that are supported in either the Bayesian (posterior probabilities, BPP) and Maximum likelihood (ML bootstrap percentage, MLB) analyses are indicated. BPP values (in bold) above 0.70 and MLB values above 50% are given above/below branches. The newly sequenced collection is in bold.

**Figure 2.**
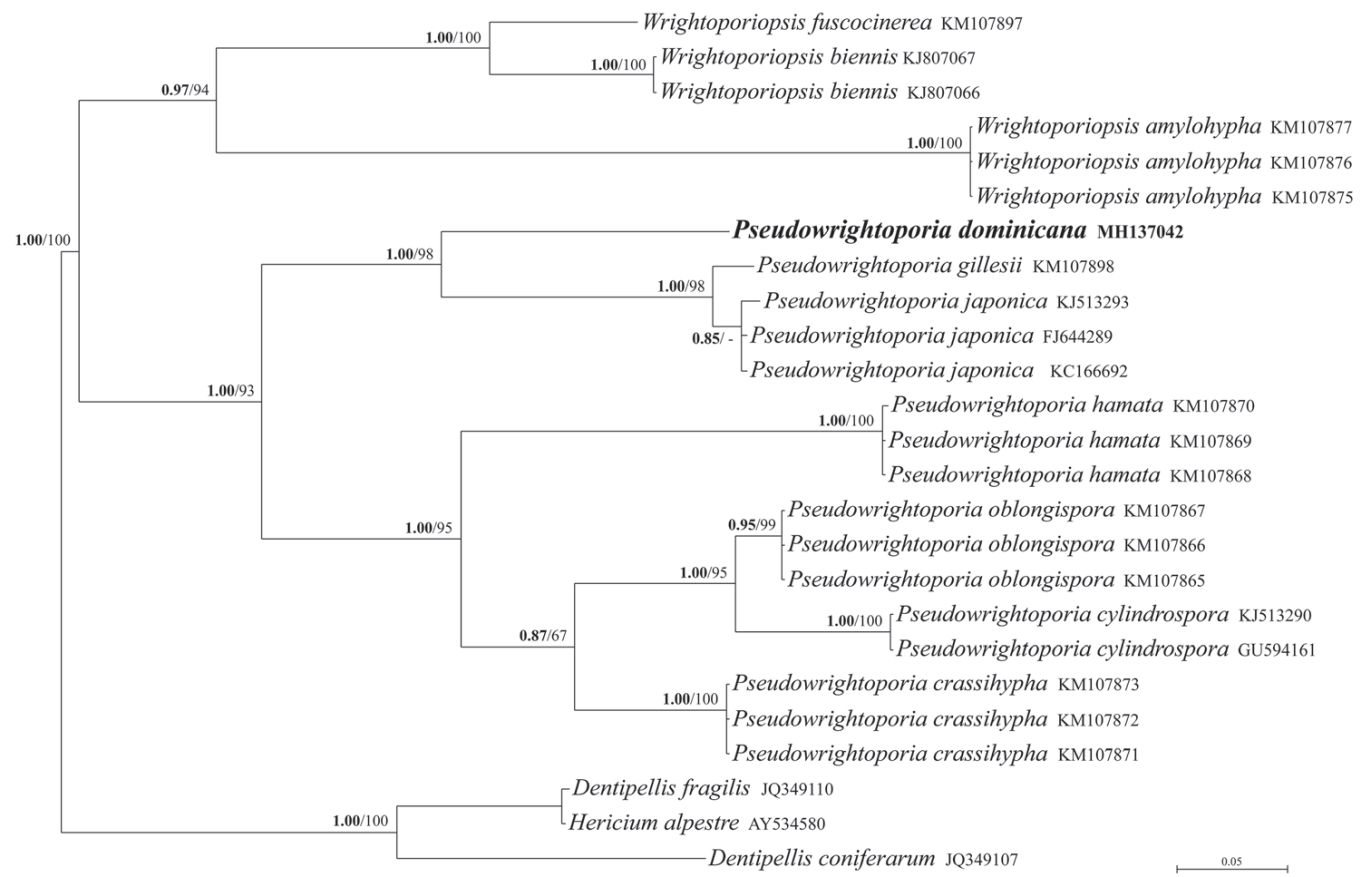
Bayesian phylogram obtained from the nrITS sequence alignment of *Pseudowrightoporia* and *Wrightoporiopsis* species. *Dentipellis coniferarum*, *D. fragilis* and *Hericium alpestre* were used as outgroup taxa. Values for clades that are supported in either the Bayesian (posterior probabilities, BPP) and maximum likelihood (ML bootstrap percentage, MLB) analyses are indicated. BPP values (in bold) above 0.70 and MLB values above 50% are given above/below branches. The newly sequenced collection is in bold.

Genomic DNA was isolated from 10 mg of a dried voucher specimen (JBSD 127410), using the DNeasy Plant Mini Kit (Qiagen, Milan) according to the manufacturer’s instructions. Primers LR0R/LR6 (Vilgalys and Hester 1990, Vilgalys lab. http://www.botany.duke.edu/fungi/mycolab) were used for the nrLSU (28S) DNA amplification and universal primers ITS1F/ITS4 for the ITS region amplification (White et al. 1990, Gardes and Bruns 1993). Amplification reactions were performed in a PE9700 thermal cycler (Perkin-Elmer, Applied Biosystems, Norwalk) in 25 ml reaction mixtures using the following final concentrations or total amounts: 5 ng DNA, 1 × PCR buffer (20 mM Tris/HCl pH 8.4, 50 mM KCl), 1 mM of each primer, 2.5 mM MgCl_2_, 0.25 mM of each dNTP, 0.5 unit of Taq polymerase (Promega, Madison). The PCR programme was as follows: 3 min at 95 °C for 1 cycle; 30 s at 94 °C, 45 s at 50 °C, 2 min at 72 °C for 35 cycles, 10 min at 72 °C for 1 cycle. PCR products were resolved on a 1% agarose gel and visualised by staining with ethidium bromide. The PCR products were purified with the AMPure XP kit (Beckman Coulter, Pasadena) and sequenced by MACROGEN (Seoul). The sequences were submitted to GenBank (http://www.ncbi.nlm.nih.gov/genbank/) and their accession numbers are reported in Figs 1–2.

### Sequence alignment, dataset assembly and phylogenetic analysis

Sequences were checked and assembled with Geneious 5.3 (Drummond et al. 2010) and compared to those available in the GenBank database (http://www.ncbi.nlm.nih.gov/Genbank/) using the BLASTN algorithm (Altschul et al. 1990). Based on BLASTN results, sequences were selected according to the recent monographic work on *Wrightoporia* s.l. by Chen et al. (2016).

Two phylogenetic analyses were performed: the first, based on a combined nrITS and nrLSU sequences dataset, to focus on the phylogenetic position of the new species in the Russulales (Russuloid clade); the second, based only on a nrITS dataset was restricted to the taxa closely related to *P. dominicana* according with the previous combined data analysis. Alignments were generated for each nrITS and nrLSU dataset using MAFFT (Katoh et al. 2002) with default conditions for gap openings and gap extension penalties. The two alignments were imported into MEGA 6 (Tamura et al. 2013) for manual adjustment. The best-fit substitution model for each single alignment was estimated by both the Akaike information criterion (AIC) and the Bayesian information criterion (BIC) with jModelTest 2 (Darriba et al. 2012). The GTR + G model was chosen for both the nrITS and nrLSU alignments. The sequences of *Sistotrema brinkmannii*, *S. coronilla*, *S. muscicola* and *S. sernanderi* were used as outgroup taxa (Larsson and Larsson 2003, Chen et al. 2016) in the combined analysis; *Dentipellis coniferarum*, *D. fragilis* and *Hericium alpestre* were selected as outgroup taxa in the nrITS analysis. The ITS dataset was not partitioned into ITS1, 5.8S and ITS2 subsets. Phylogenetic hypotheses were constructed under Bayesian inference (BI) and Maximum likelihood (ML) criteria. The BI was performed with MrBayes 3.2.6 (Ronquist et al. 2012) with one cold and three incrementally heated simultaneous Monte Carlo Markov chains (MCMC) run for 10 million generations, under the selected evolutionary model. Two simultaneous runs were performed independently. Trees were sampled every 1,000 generations, resulting in overall sampling of 10,001 trees per single run; the first 2,500 trees (25%) were discarded as burn-in. For the remaining trees of the two independent runs, a majority rule consensus tree showing all compatible partitions was computed to obtain estimates for Bayesian posterior probabilities (BPP). ML estimation was performed through RAxML 7.3.2 (Stamatakis 2006) with 1,000 bootstrap replicates using the GTRGAMMA algorithm to perform a tree inference and search for a good topology. Support values from bootstrapping runs (MLB) were mapped on the globally best tree using the “-f a” option of RAxML and “-x 12345” as a random seed to invoke the novel rapid bootstrapping algorithm. BI and ML analyses were run on the CIPRES Science Gateway web server (Miller et al. 2010). Only BPP and MLB values over 0.70 and 50%, respectively, are reported in the resulting trees (Figs 1–2). Branch lengths were estimated as mean values over the sampled trees.

## Results

The combined nrITS and nrLSU data matrix comprised 118 sequences (including 117 from GenBank) and includes 2132 positions. The nrITS data matrix comprises a total of 25 sequences (including 24 from GenBank) and includes 687 positions. As both Bayesian and Maximum likelihood analyses produced comparable topologies, only the Bayesian trees with both BPP and MLB values are shown (Figs 1–2). In the combined two-gene phylogeny of Russulales taxa (Fig. 1), the new species falls, as an independent phylogenetic branch, in the Hericiaceae within the *Pseudowrightoporia* cluster. *Pseudowrightoporia dominicana* is sister (BPP = 1.00, MLB = 95) to *P. japonica*. *Pseudowrightoporia* is shown to be sister (BPP = 1.00, MLB = 100) to a well-supported clade (BPP = 1.00, MLB = 80) consisting of *Wrightoporiopsis* and *Dentipellicula*, as previously highlighted by Chen et al. (2016). The small ITS analysis restricted to species of *Pseudowrightoporia* and *Wrightoporiopsis* (Fig. 2) supports *P. dominicana* as a new species and indicates *P. gillesii* and *P. japonica* as its phylogenetically closest species.

### Taxonomy

#### Pseudowrightoporia dominicana

Taxon classificationFungiRussulalesHericiaceae

Angelini, Losi & Vizzini sp. nov.

MB824844

Fig. 3

##### Holotype.

Dominican Republic. La Vega (Province), Jarabacoa (Municipality), Montaña (Locality), 19°06'39"N, 70°37'57"W, on an unidentified live trunk of a deciduous tree, in a mixed mountain forest with several broadleaved species and pines (*Pinus occidentalis*), 17 December 2016, Claudio Angelini, (JBSD 127410, isotype ANGE 789).

##### Etymology.

The epithet refers to the country, The Dominican Republic, where this species was found.

**Figure 3.**
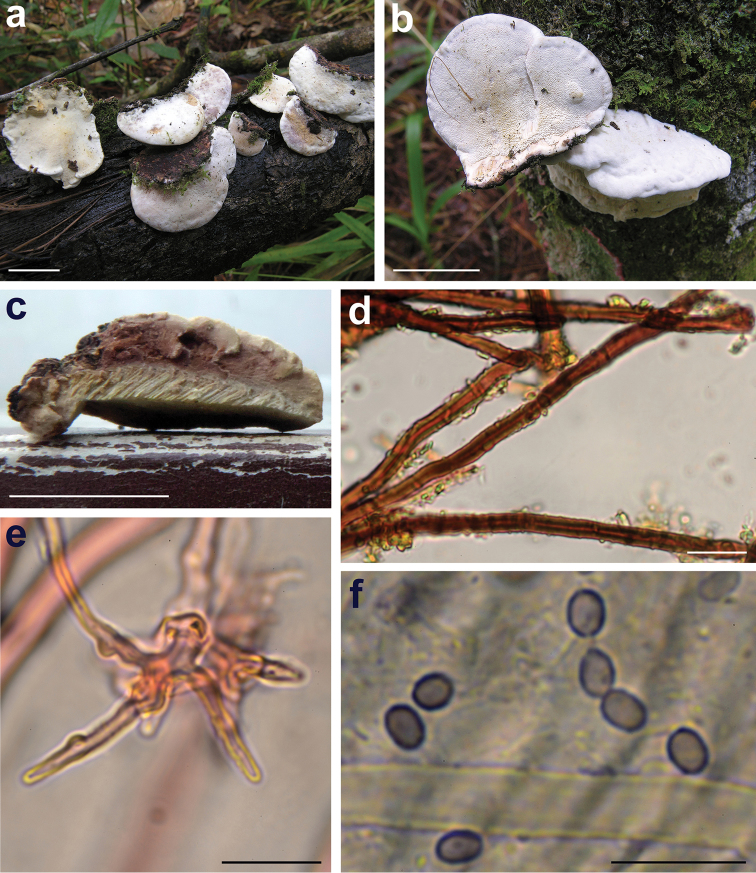
*Pseudowrightoporia dominicana* (JBSD 127410) **a, b** fresh basidiomes in situ **c** cut side of the basidiome **d** dextrinoid skeletal hyphae **e** binding hypha **f** amyloid spores. Microscopical elements observed in Melzer’s anionic reagent. Scale bars: 10 mm (**a–c**); 10 μm (**d–f**).

Basidiomata annual, pileate, sessile, single or in small clusters, fibrous-tough (Fig. 3a and b). Pileus broadly attached to dimidiate, up to 25 mm wide and 15 mm deep, 5–10 mm thick; upper surface white to cream with pinkish tint, velutinate to glabrous, azonate, smooth; margin rounded, even or slightly lobed; pore surface concolorous with the pileus surface, pores round to angular, at first cupulate, 6–8 per mm, dissepiments thick and entire; tube layer 2–4 mm thick, whitish to cream; context pinkish (Fig. 3c), homogenous, tough-fibrous, up to 6 mm thick. Hyphal system di-trimitic; generative hyphae clamped, hyaline, thin-walled, 2.2–4.8 µm wide; skeletal hyphae thick-walled, rarely branched, 2.4–5.6 µm wide, dextrinoid especially in the trama (Fig. 3d); contextual binding hyphae thick-walled, short-branched, 1.6–2.4 µm wide, weakly dextrinoid (Fig. 3e). Cystidia none. Basidia densely united, clavate, 4-sterigmate, 8–12 × 4–5 µm. Basidiospores (2.6–)2.98–*3.2*–3.43(–3.6) × (1.8–)1.96–*2.2*–2.44(–2.8) µm (n = 40), Q = (1.14–)1.28–*1.44*–1.6(–1.89), broadly ellipsoid to ellipsoid, finely asperulate, thin- to slightly thick-walled, distinctly amyloid (Fig. 3f).

##### Habit, habitat and distribution.

Pileate, gregarious on a live trunk of deciduous tree, so far known only from the type locality.

## Discussion

All the phylogenetic analyses show *P. dominicana* to be a distinct lineage in the genus *Pseudowrightoporia* (Figs 1–2). The new species displays a unique combination of outstanding characters such as pileate basidiomes, pink context, very small spores and di-trimitic hyphal system (Fig. 3). In particular, the presence of binding hyphae (only in the context) is quite unusual in *Pseudowrightoporia* as well as in the other genera of *Wrightoporia* s.l. (Ryvarden 1982, 1987, 2000, 2016; David and Rajchenberg 1987, Dai 1995, Núñez and Ryvarden 2001, Dai and Cui 2006, Hattori 2008, Chen and Cui 2012, 2014; Chen and Yu 2012, Jang et al. 2013, Westphalen et al. 2014, Chen et al. 2016, Drechsler-Santos et al. 2016, Campi et al. 2017); binding hyphae have so far been reported only in *P. aurantipora* (Hattori 2008), *W. brunneo-ochracea* A. David & Rajchenb. (David and Rajchenberg 1985), *W. trimitica* (Corner) Stalpers (Corner 1989, Stalpers 1996) and *Larssoniporia tropicalis* (Cooke) Y.C. Dai, Jia J. Chen & B.K. Cui, (Núñez and Ryvarden 2001).

*Pseudowrightoporia gillesii* and *P. japonica* are the species phylogenetically most closely related to *P. dominicana* (Figs 1–2). *Pseudowrightoporia gillesii*, originally described from Africa (Gabon), is characterised by an effused-reflexed basidiome, chestnut ochraceous context, dimitic context, skeletal hyphae dextrinoid only in the pore mouths and presence of lageniform to mucronate cystidiola (David and Rajchenberg 1987). *Pseudowrightoporia japonica* (= *Wrightoporia luteola* B.K. Cui & Y.C. Dai according with Jang et al. 2013 and Chen et al. 2016) shows a basidiome shape ranging from pileate (and then with a zoned pileus) to resupinate, a pore surface cream to wood-coloured, a dimitic hyphal system and more elongated spores, up to 4 × 2.6 µm (Núñez and Ryvarden 1999, 2001; Jang et al. 2013).

Amongst the morphologically most similar species to *P. dominicana*, *Wrightoporia dimidiata* A. David & Rajchenb. from Asia (Singapore) is distinguished by a hymenophore with 3–4 pores per mm, dimitic hyphal system, spores measuring 3.5–4 × 3 µm and presence of cystidiola, gloeocystidia and gloeopleurous hyphae (David and Rajchenberg 1987). From above, the new species may resemble the pileate basidiomes of *Wrightoporia cremea* Ryvarden from Brazil, but the latter has larger pores (3–4 per mm) and spores (subglobose, 3–4 µm in diam.), dimitic hyphal system, in addition to a cream to pale ochre context (Ryvarden 1987, 2017 and pers. comm.). Finally, *P. aurantipora* from Japan, *W. brunneo-ochracea* from Guadeloupe, *W. trimitica* from Malaya and the pantropical *W. tropicalis* share with *P. dominicana* the presence of binding hyphae, but *P. aurantipora* differs in having resupinate basidiomes with light orange to brown orange 4–6/mm pores, context orange without pinkish hues, tramal skeletal hyphae strongly covered with granules near the tip and longer spores, 3–4.2 × 2–3 μm (Hattori 2008); *W. brunneo-ochracea* differs in having effused-reflexed basidiomes with ochraceous, irregular to angular pores, 3–4 per mm, a thin ochraceous context, non-dextrinoid skeletal hyphae and narrower spores, 3–3.5 × 2 μm (David and Rajchenberg 1985, Ryvarden 2016); *W. trimitica* has dimidiate basidiomes, with a short resupinate foot, ochraceous to wood-coloured pores and up to 4 μm long spores (Corner 1989, Stalpers 1996); *Larssoniporia tropicalis* has resupinate, applanate to pulvinate, widely effused, grey to black perennial and very woody basidiomes, grey to brown pore surface, thick-walled and heavily enrsusted cystidia, blunt at the apex, presence of gloeocystidia and subglobose spores 3–4 × 2–3 μm (Ryvarden and Johansen 1980, Núñez and Ryvarden 2001, Ryvarden 2016).

## Supplementary Material

XML Treatment for Pseudowrightoporia dominicana
